# Crucial choices in a global health crisis: Revealing the demand and willingness to pay for a hypothetical monkeypox vaccine – the PREVENT study

**DOI:** 10.7189/jogh.13.04033

**Published:** 2023-05-05

**Authors:** Bach Xuan Tran, Linh Anh Do, Thao Phuong Hoang, Laurent Boyer, Pascal Auquier, Guillaume Fond, Huong Thi Le, Minh Ngoc Le Vu, Trang Huyen Thi Dang, Anh Hai Tran Nguyen, Carl A Latkin, Roger CM Ho, Cyrus SH Ho, Melvyn WB Zhang

**Affiliations:** 1 Institute for Preventive Medicine and Public Health, Hanoi Medical University, Hanoi, Vietnam; 2 Research Centre on Health Services and Quality of Life, Aix Marseille University, Marseille, France; 3 SC Johnson College of Business, Cornell University, Ithaca, New York, USA; 4 ActionAid Vietnam, Hanoi, Vietnam; 5 University of Medicine and Pharmacy, Vietnam National University, Hanoi, Vietnam; 6 Bloomberg School of Public Health, Johns Hopkins University, Baltimore, Maryland, USA; 7 Department of Psychological Medicine, Yong Loo Lin School of Medicine, National University of Singapore, Singapore, Singapore; 8 Institute for Health Innovation and Technology (iHealthtech), National University of Singapore, Singapore, Singapore; 9 Lee Kong Chian School of Medicine, Nanyang Technological University Singapore, Singapore, Singapore

## Abstract

**Background:**

The latent monkeypox outbreak has become the most emergent public health challenge globally. This study was conducted to assess the acceptability, and willingness to take and pay for a hypothetical Monkeypox vaccine among the Vietnamese general public as well as investigate preference for individual vaccine attributes.

**Methods:**

An online cross-sectional study was conducted using snowball sampling among 842 respondents in Vietnam in 2022. A Discrete choice experiment (DCE) on preference for six major attributes of vaccine: effectiveness, immunity duration, side effects, mortality rate, restriction, and the cost was applied.

**Results:**

Fear of the impact of monkeypox on public health and the economy, vaccine service satisfaction and responsibility to the community were the most weighted factors in the decision to take a hypothetical monkeypox vaccine. Two-thirds of participants were willing to take the vaccine, while insufficient information on monkeypox and the vaccine were the main reasons for vaccine hesitancy. For vaccine attributes, the mortality rate after seven days of vaccination was the most weighted while cost was the least influential attribute. Factors associated with willingness to take and to pay for the monkeypox vaccine included knowledge of transmission, geographical location, service satisfaction, and risk of infection, while financial burden and fear of vaccine were major drivers of hesitancy.

**Conclusion:**

Our findings underline an urgent need for effective information dissemination through social media and counseling. The implementation of nationwide monkeypox vaccination requires prioritization and support for high-risk groups as well as consideration for the country’s financial resources.

In July 2022, the World Health Organization declared monkeypox a global health emergency [1-3]. Mass vaccination is a prioritized response to the latent monkeypox pandemic, given that over 70% of the world’s population has lost immunity against smallpox and, consequently monkeypox [2]. However, unlike COVID-19, monkeypox is not an entirely novel disease, and a preventive vaccine is available for the general population. Controversies about the COVID-19 vaccine have largely impacted the acceptance of the current monkeypox vaccine, specifically in heightened wariness about long-term side effects and immunity duration. Common concerns include the risk of autoinoculation, disseminated infection, and post-vaccination encephalitis [1]. Therefore, understanding public preference for the monkeypox vaccine is central to the development and implementation of vaccination programs.

The monkeypox epidemic that emerged during the COVID-19 pandemic resurged in many settings due to insufficient vaccination and boosting coverage [4]. This global experience emphasizes the importance of introducing a new monkeypox vaccine, which must be not only effective and accepted but also timely. From a public health perspective, it is critical to ensure the necessary infrastructure for service delivery and encourage access to and effective use of vaccines by promoting awareness, acceptance, readiness, and willingness to take the vaccine among the public. Given the novelty of the monkeypox outbreak to date, there is little evidence of the willingness to receive and pay for a new monkeypox vaccine and suggestions of monkeypox vaccination hesitancy in the context of the COVID-19 pandemic. Few studies demonstrated that the younger group, having knowledge and perception of disease severity, and receiving health warnings were substantially correlated with lower monkeypox vaccine hesitancy [5,6].

Besides empirical evidence on social and structural barriers to vaccination, frameworks for understanding vaccination motivation are needed to assess willingness to receive a monkeypox vaccine. For instance, the integrated model of the Health Belief Model and Theory of Reasoned Action identified behavioral determinants such as benefits, limitations, attitude, and subjective norms to be correlated with vaccine acceptance [7,8]. Meanwhile, a more detailed and pandemic-specific model, the World Health Organization increasing vaccination model, defines perception, social conformism, confidence in the vaccine, and convenience in accessing a vaccine as some of the most significant determinants of vaccination willingness [9,10]. Although existing frameworks have been updated extensively to adapt to new health emergencies, the economic aspects of these frameworks are lacking which is, however, very important for policy implications in resource-scare settings. In addition, one’s decision to take a monkeypox vaccine is more likely to be influenced by personal preference for known vaccine attributes rather than the mere perception of severity, benefits, and limitations as suggested by conventional frameworks. This change in vaccine perception means that simply evaluating attributes independently is not adequate to conclude public preference for a vaccine; instead, several attributes should be evaluated simultaneously under different scenarios to understand the complex decision process by specific beneficial groups. Discrete choice experiments (DCEs) are a useful technique to assess individual preference by choosing several hypothetical alternatives with different attributes. DCEs have their theoretical underpinning in the Random Utility Model, which models the choices of individuals among discrete sets of alternatives and describes the preferences by a utility function. Personal choice, or preference, is the alternative with the highest perceived utility [11]. The application of DCEs is evident in health services and policy research as well as studies of vaccination preferences but is underused in resource-scare settings, given their complexity in the study design and implementation [12]. This study aimed to assess the knowledge of the general Vietnamese population on monkeypox and evaluate their willingness to take and pay for a hypothetical monkeypox vaccine.

## METHODS

### Study design

The Preference for Vaccine Evaluation & Trail (The PREVENT Study) is a Vietnam nationwide assessment using a patient-centered design to inform health technology development and acceleration. Designed using the Qualtrics system (www.qualtrics.com), the PREVENT study included an online interactive questionnaire to target subjects across different regions of Vietnam from April to August 2022. Eligibility requirements included being Vietnamese living in Vietnam, aged 16 and above, being referred, and agreeing to complete the survey. The snowball sampling method [13] was used for disseminating the survey, involving 20 seeders who were first chosen respondents in all three regions: Northern, Central, and Southern. These seeders were asked to participate in the survey and then refer it to their peers. Participants took approximately 30 minutes to complete the questionnaire and then were encouraged to introduce more acquaintances and colleagues to participate in the survey. Participants were informed of the benefits and risks of participating and gave informed consent by ticking the box at the top of the questionnaire. Records were monitored and tracked using IP address by Qualtrics system to avoid duplicates and ensure the validity of the data set, then extracted, analyzed, and stored safely and confidently and used merely for research purposes.

The PREVENT Study included five topics of interest, namely: 1) monkeypox, 2) COVID-19 vaccine booster for adults, 3) COVID-19 vaccine for children, 4) HIV vaccine, and 5) a hypothetical pandemic in the future. After answering general social demographic questions, each respondent was randomly assigned to one of these five topics, generating five separate data sets. In total, 5700 respondents were included in the PREVENT study. The sub-study of the monkeypox vaccine included 842 complete records, and the response rate was 80.6%.

### Measurement and instrument

We applied a standard procedure for generating the research instrument. Initially, a literature review was conducted to identify gaps in knowledge and important facets that have emerged from previous studies. Second, we constructed the questionnaire, covering the breadth of measurement of interest. A group of experts in public health, infectious diseases, health services, econometrics, linguistics, representatives of target groups, and research assistants jointly deliberated throughout the process of translating, rephrasing, piloting, and shorting the questionnaire. Finally, the tool included five major sessions: 1) socio-demographics, 2) history of COVID-19 infection and vaccination, 3) knowledge and perception of monkeypox (15 items in three domains), 4) willingness to take a hypothetical new monkeypox vaccine, and 5) willingness to pay for it. The details questionnaire, construction, and items are presented in Section 1 in the **Online Supplementary Document**.

### Discrete choice experiment (DCE)

Six major drivers of individual choices on vaccination were derived from a thorough review of the literature, namely 1) effectiveness of the vaccine, 2) immunity duration, 3) side effects, 4) mortality rate within seven days after vaccination, 5) restriction if not vaccinated, and 6) costs. These attributes were then assigned two-five levels for choosing, contributing to a total of 3^1^ × 4^1^ × 2^3^ × 5^1^ = 480 possible alternatives (**Table 1**). Each participant was asked to respond to seven different scenarios based on generated combinations. The sample size was estimated to be 357 per group (Section 2 in the **Online Supplementary Document**).

**Table 1:** Vaccine attributes in Discrete choice experiment

Attributes			Options		
Effectiveness	<60%	60%-90%	>90%		
Immunity duration	3-6 months	6-12 months	1-3 year	Lifetime	
Side effects	Light, normal activities	Severe, fatigue, immobility			
Mortality rate after vaccinated	1 / 100 000	10 / 100 000			
Restriction if not vaccinated	No restrictions	Cannot go to crowded places			
Cost*	100 000 VND	200 000 VND	500 000 VND	1 000 000 VND	2 000 000 VND

VND – Vietnamese đồng

*1 US dollar (US$) = 23 000 VND.

### Statistical analysis

Statistical analysis was performed using Qualtrics and STATA software (Stata 15. version 15. StataCorp; 2017). With missing data, we used the Listwise Deletion method to clean data before analyzing it. The Listwise Deletion method handles missing values and removes empty observations of all variables.

The internal consistency reliability was assessed using Cronbach’s alpha. The Cronbach’s alpha value of 0.7 or above was considered acceptable. We also assessed domain-domain correlation, item-item correlation, item-total correlation, and Cronbach’s alpha of the domain if the item was deleted.

The Exploratory Factor Analysis (EFA) using principal component analysis (PCA) was performed to evaluate the optimal structural model of the instrument according to the observed data. The number of factors was determined based on the Scree plot, and parallel analysis, along with eigenvalues and the proportion of variance explained. Items with a loading value ≥0.4 were included in the relevant component. Hence, in terms of the knowledge and perception of monkeypox, there were three domains, including general knowledge about monkeypox (three items), knowledge of transmission routes and ways to prevent monkeypox (nine items), and incorrect knowledge about the route of transmission, and treatment drugs of monkeypox (three items).

Potential covariates for full models of the decision to take and willingness to pay for the monkeypox vaccine are socioeconomic status, COVID-19 characteristics, related information regarding the COVID-19 vaccine, factors affecting intention to vaccinate for diseases prevention, interpersonal factors, and monkeypox's knowledge and perception. We used Ordered Logistic Regression to identify factors related to the decision to take and willingness to pay for the monkeypox vaccine. The number of observations was 705 with the Pseudo R2 was 0.0790 and 0.0684. The *P*-value *P* < 0.05 was considered statistically significant.

In DCE data analysis, individual-based utility models were yielded using Hierarchical Bayes estimation that uses Bayesian methods to probabilistically derive the relative value of each tested variable.

## RESULTS

The majority of our sample were females and aged from 16 to 24 years old. The most common education level is bachelor, followed by a mean average monthly income per household of 3-10 million Vietnamese đồng (VND) (**Table 2**). 62.7% had been infected with COVID-19, mostly in the recent 3-6 months. The majority of our sample also received the mandatory and booster doses of the COVID-19 vaccine. In terms of factors affecting vaccination to prevent disease, the mean score of “concerns about the impact and composition of the vaccine”, “fear of vaccine”, and “responsibility to the community” was 7.0 (standard deviation (SD) = 2.0), 5.9, (SD = 2.4) and 7.1 (SD = 2.6). Regarding interpersonal factors, the average score of “risks of infected diseases”, “fear of the impact of the disease on health and economy”, and “service satisfaction” were 6.8 (SD = 2.0), 7.8 (SD = 1.9) and 7.3 (SD = 1.7), respectively (**Table 3**). The willingness to receive the monkeypox vaccine was positive in the majority of our sample. Among the 1.5% who were unwilling to get vaccinated for monkeypox, the main concerns were insufficient information about monkeypox (41.2%) and the vaccine’s side effects (32.6%). Vaccine cost was also an economic burden for 63.4% of participants (**Table 4**).

**Table 2:** Demographic characteristics of participants

Characteristics	n	%
Gender		
*Male*	239	28.6
*Female*	595	71.4
Age group		
*16-19*	298	35.4
*20-24*	345	41.0
*>25*	198	23.5
Educational attainment		
*Not graduated from high school*	92	11.0
*Graduated from high school*	65	7.7
*College / university / postgraduate*	683	81.3
Marital status		
*Single / divorced / widowed*	691	82.3
*Married*	149	17.7
Occupation		
*Healthcare worker / medical students*	357	42.5
*Other students*	219	26.1
*Other occupation*	264	31.4
Children status		
*No children yet*	700	83.6
*Pregnant / have children*	137	16.4
Monthly household income per capita		
Under 1 million VND*	233	28.9
1-3 million VND*	154	19.1
3-10 million VND*	315	39.1
>10 million VND*	104	12.9
Area		
*Hanoi – Capital city*	335	42.0
*Northern Provinces / cities*	108	13.5
*Southern Provinces / cities*	114	14.3
*Central and Central Highlands*	174	21.8
*Other provinces*	67	8.4
Total	842	

VND – Vietnamese đồng

*1 US dollar (US$) = 23 000 VND.

**Table 3:** Health characteristics regarding COVID-19, factors affecting vaccination to prevent diseases in general and Interpersonal factors

Characteristics	n	%
Personal and family history of COVID-19		
*Nobody has ever had COVID-19*	117	13.9
*You had COVID-19*	528	62.7
*Have adults at home with COVID-19*	456	54.2
*Children <18 years old at home with COVID-19*	227	27.0
Time since the last COVID-19 infection		
*Not yet infected*	264	31.7
*1-3 months*	184	22.1
*3-6 months*	333	40.0
*>6 months*	51	6.1
Health status		
*Completely healthy (100%)*	342	40.6
*Relatively healthy (80 -<100%)*	388	46.1
*Compromised / with illness*	112	13.3
History of COVID-19 vaccination of individual and family		
*Two injections*	225	26.7
*More than three injections / boosters*	559	66.4
*At least one injection for children below 12 years old*	57	6.8
*At least one injection for children from 12-17 years old*	92	10.9
*At least two injections for adults*	401	47.6
	**Mean**	**SD**
Factors affecting vaccination to prevent disease		
*Concerns about the impact and composition of the vaccine (range 1-10)*	7.0	2.0
*Fear of vaccine (range 1-10)*	5.9	2.4
*Responsibility to the community (range 1-10)*	7.1	2.6
Interpersonal factors		
*Risks of infected diseases (range 1-10)*	6.8	2.0
*Fear of the impact of the disease on health and economy (range 1-10)*	7.8	1.9
*Service satisfaction (range 1-10)*	7.3	1.7

**Table 4:** Willing to inject and pay for vaccine characteristics of respondents

Characteristics	n	%
Willingness to injection		
*Will not vaccinate*	13	1.5
*Not decided yet, waiting for more information*	111	13.2
*Will vaccine once vaccine becomes officially approved*	551	65.4
*Ready to vaccinate in the vaccine trial*	167	19.8
Reasons for vaccine hesitancy		
*Insufficient information about monkeypox*	296	41.2
*Not feeling well*	61	8.5
*Having underlying diseases / comorbidities*	27	3.8
*Allergic*	42	5.9
*Insufficient information about vaccine’s side effects*	234	32.6
*Not decided, waiting for more information*	58	8.1
*Cannot access health institutions to vaccinate*	22	3.1
*Still suffering from side effects of last vaccination*	30	4.2
*Feeling vaccination is unnecessary*	26	3.6
Willing to pay		
*Unwilling to pay*	162	19.2
*20% of cost*	124	14.7
*50% of cost*	258	30.6
*80% of cost*	122	14.5
*Full cost*	176	20.9
Economic burden		
*No*	302	36.6
*Yes*	522	63.4

The EFA results about monkeypox's knowledge and perception included three factors. 1) General knowledge about monkeypox: mean (m) = 6.82 (SD = 4.18), Cronbach’s alpha = 0.90; 2) knowledge of transmission routes and ways to prevent monkeypox: m = 4.34 (SD = 3.31), Cronbach’s alpha = 0.86; 3) misconception on monkeypox: m = 1.30 (SD = 2.7) and Cronbach’s alpha = 0.75 (**Table 5**).

**Table 5:** Exploratory factor analysis of knowledge and perception of monkeypox among participants

Items	n (%)	General knowledge about monkeypox	Knowledge of transmission routes and ways to prevent monkeypox	Misconception on monkeypox
Monkeypox only exists in Western and Middle Africa	814 (79.6)	0.5973		
Vietnam is at risk of developing a monkeypox outbreak	648 (63.4)		0.4256	
Monkeypox is spreading fast globally	626 (61.2)		0.6014	
It is difficult to spread monkeypox between humans	208 (20.3)			0.7706
Monkeypox is spread through sexual means	651 (63.7)	0.9087		
Monkeypox is spread through skin contact and droplets	465 (45.5)		0.6168	
Monkeypox has the same manifestation as smallpox	383 (37.5)		0.5136	
Having multiple pustules is a symptom of monkeypox	346 (33.9)		0.5822	
Fever, headache, swollen lymph nodes are symptoms of monkeypox	460 (45.0)		0.6254	
Monkeypox can result in fatigue and death	374 (36.6)		0.623	
Treatment for monkeypox is available	70 (6.8)			0.4476
Not eating wild animals is a prevention means	318 (31.1)		0.5922	
Reducing interactions with wild animals is a prevention means	378 (37.0)		0.6495	
Monkeypox is not transmitted through handshakes, hugs, and kisses	121 (11.8)			0.7863
Strengthening personal hygiene and nutritional plan are prevention means	625 (61.1)	0.9104		
Reliability (Cronbach’s alpha)		0.89	0.86	0.75
Score (range 0-10), mean (SD)		6.14 (1.40)	4.54 (3.30)	1.40 (2.80)

SD – standard deviation

Participants who were female and married had a higher willingness to take the monkeypox vaccine. Participants who resided in the South had a lower willingness to take than those from Hanoi, while residents in Central and Central Highlands had higher vaccine acceptance and willingness to pay. Participants who had higher risks of infected diseases and service satisfaction scores had a higher level of willingness to inject the vaccine. A higher score of knowledge of transmission routes and ways to prevent monkeypox and satisfaction with service were positive factors associated with willingness to pay for the monkeypox vaccine. By contrast, a higher score of fear of the vaccine and the burden of medical expenses were likely to be associated with a lower level of willingness to pay for monkeypox vaccine (**Table 6**).

**Table 6:** Factors associated with willingness to take and willingness to pay for monkeypox vaccine among participants

Characteristics	Willingness to take		Willingness to pay	
	**(1 “not inject” – 4 “willing to participate in trials”)**		**(1 “no pay” – 5 “100% fee”)**	
	**OR**	**95% CI**	**OR**	**95% CI**
Socio-economic				
Gender (female vs. male – Ref.)	0.50*	0.34-0.73		
Marital status (married vs. single / divorced / widowed – Ref.)	0.57†	0.36-0.92		
Monthly household income per capita (<1 million VND – Ref.)				
*1-3 million VND*	1.22	0.77-1.92	1.79*	1.21-2.64
*3-10 million VND*	0.71‡	0.48-1.07	1.43†	1.03-2.00
*>10 million VND or above*	0.69	0.39-1.22	4.57*	2.77-7.54
Location (vs. Hanoi – Ref.)				
*Northern provinces*	1.41	0.84-2.35	1.34	0.88-2.04
*Southern provinces*	0.60†	0.37-0.96	1.36	0.91-2.03
*Central and Central Highlands*	1.57†	1.02-2.41	1.85*	1.29-2.66
*Other provinces*	0.88	0.46-1.68	0.79	0.47-1.34
Health status (vs. Completely healthy 100% – Ref)				
*Relatively healthy (80 -<100%)*	0.64†	0.45-0.91	0.76‡	0.56-1.04
*Compromised/with illness*	0.47*	0.28-0.79	0.61†	0.40-0.95
History of COVID-19 vaccination of self and family				
*Had mandatory two injections*			1.36†	1.00-1.85
*At least two injections for adults*	0.71†	0.51-0.98		
Knowledge and perception of monkeypox				
*Knowledge of transmission routes and ways to prevent monkeypox (unit: score)*			1.04†	1.00-1.09
Factors affecting vaccination to prevent disease				
*Fear of vaccine (unit: score)*			0.95*	0.93-0.97
Interpersonal factors				
*Risks of infected diseases (unit: score)*	1.06*	1.02-1.09		
*Fear of the impact of the disease on health and economy (unit: score)*	0.93*	0.90-0.97		
*Service Satisfaction (unit: score)*	1.04†	1.00-1.09	1.06*	1.03-1.09
Economic burden (yes vs. no – Ref)	0.76	0.54-1.07	0.46*	0.34-0.61

OR – odds ratio, CI – confidence interval

*P < 0.01, †*P* < 0.05, ‡*P* < 0.1.

**Figure 1:**
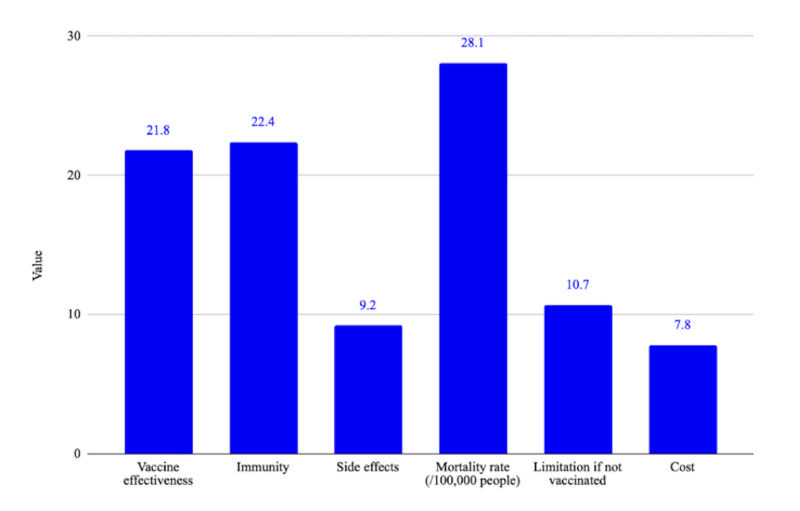
Feature importance of vaccine attributes during the decision-making process.

Our DCE result suggested that mortality rate had the most influence on the decision-making process, followed by immunity and vaccine effectiveness. The cost was the least weighted attribute at 7.8 points (**Figure 1**). The best scenario for respondents included above 90% effectiveness with life-long immunity, minor side effects, low mortality rate, and no limitation. For this scenario, the mean willingness to pay among participants was 200 000 VND. The actual vaccine package based on the current smallpox vaccine included: 60%-90% effectiveness, life-long immunity, potential adverse side effects such as fatigue, fever or blister, insignificant mortality rate, and few limitations if not vaccinated. For this package, the generated willingness to pay was 187 000 VND (**Table 7** and **Table 8**).

**Table 7:** The optimal monkeypox vaccine attributes package

**Vaccine effectiveness**	More than 90%
**Immunity**	Life-long
**Side effects**	Minor, do not affect daily activities
**Mortality rate ( / 100 000 people)**	One death
**Limitation if not vaccinated**	No
Cost*	200 000 VND

VND – Vietnamese đồng

*1 US dollar (US$) = 23 000 VND.

**Table 8:** The actual vaccine package based on the current smallpox vaccine

**Vaccine effectiveness**	60%-90%
**Immunity**	Life-long
**Side effects**	Severe, fatigue and immobility
**Mortality rate ( / 100 000 people)**	One death
**Limitation if not vaccinated**	No
Cost*	187 000 VND

VND – Vietnamese đồng

*1 US dollar (US$) = 23 000 VND.

## DISCUSSION

Our findings indicate a high willingness to take monkeypox vaccines and a moderate willingness to pay, provided that sufficient information about the pandemic and vaccine characteristics are available; this preference was the same among the general population and health professionals / medical students. While most participants had a basic understanding of monkeypox, more information should be given on monkeypox symptoms and transmission. Drivers of vaccine decisions were fear of the impact on community health and economy, satisfaction with health and vaccination services, sense of responsibility to the community, and concerns about the composition of the vaccine. Willingness to take and to pay for the vaccines vary not only with socio-economic characteristics and vaccine attributes but also with the history of personal experience with COVID-19.

Overall, while the basic knowledge of smallpox, such as the current situation and basic symptoms, was reported as above average, our participants demonstrated a poor comprehension of monkeypox; this is consistent with recent studies on monkeypox. In fact, Indonesian general health practitioners recorded only 9% with a thorough understanding of the disease, while 23.1% mistook antibiotics as a required monkeypox treatment [5,14]. Critical public misperception can be attributed to the ineffective dissemination of monkeypox information. Indeed, more than 20% of our sample were unaware of human-to-human monkeypox transmission, nor believed that monkeypox could transmit through intimacy. In addition, among all predictors of vaccine hesitancy identified in this study and available literature, concerns about the composition of the monkeypox vaccine were consistently ranked as the most important [5,15]. Compared to COVID-19, monkeypox information was significantly less effectively communicated to the public. Although the current state of monkeypox is comparable to that of COVID-19 in January 2020, monkeypox's basic information was significantly less effectively communicated to the public than COVID-19's, which was correctly perceived by up to 86% of the population and broadcasted intensively worldwide at that time [16,17]. Informing the public about the severity and basic symptoms of monkeypox is critically important even when clinical information is not yet official, as delays in early communication had resulted in a failure to control the disease in other recent outbreaks [18].

Although the monkeypox vaccine was widely accepted, just 20% of respondents were willing to pay for it, even when people had knowledge about the disease and the related risks. Since the modern monkeypox vaccine is not yet available, the willingness to pay was determined based on smallpox vaccine attributes, which included a level of effectiveness below 90%, a life-long immunity, potential adverse side effects such as fatigue, fever or blister, and insignificant mortality rate [19,20]. For this vaccine package, participants were willing to pay 187 000 VND (8US$), which amounted to only 3.7% of the national monthly household income per capita of approximately 5 million VND (US$212). Furthermore, Vietnamese’ willingness to pay was only one-fifth of the willing to pay (WTP) recorded in Indonesia, a middle-income peer. The WTP calculated in a cross-sectional study among Indonesian frontline physicians was US$37 on average, equivalent to 19.2% of their national monthly household income per capita [21,22]. Compared to COVID-19, the WTP recorded in this study is also one-half of the WTP recorded for the COVID-19 vaccine in Vietnam in 2021 [23].

Our results also demonstrated cost as the least influential factor when making vaccine decisions compared to other clinical attributes, where all factors were weighted at least twice as much cost. Also, there was a strong correlation between satisfaction with vaccination services during COVID-19 and willingness to take and pay for the monkeypox vaccine. In terms of geographical difference, despite having a lower average income, participants in the Central and Central Highlands were more willing to take and pay for the vaccine than those in Hanoi and other major provinces. A possible explanation is that residents in rural areas were generally more physically and financially impacted by the COVID-19 pandemic, which resulted in heightened wariness to the prevalent pandemic. As rural communities also have smaller populations, it is more convenient to encourage adherence to government-led social measures, such as taking a monkeypox vaccine [24,25].

The most important implication of our findings for government intervention design emphasizes on effective communication campaigns and enhanced service accessibility. Transparent communication is needed about monkeypox presentation, risks, recommendations of clinicians, and the active role of the population in prevention and management. Given the increasing popularity of online platforms, information should be dispensed through social media as well. In the interest of early communication, public figures and influential individuals should be recruited or encouraged to raise awareness about the disease, especially in the domains where monkeypox substantially differs from COVID-19, such as transmission mode and symptoms. Social listening insights on public perception and misinformation should be more widely utilized to inform risk communication and identify areas that need addressing, such as the potential stigmatization of LGBTQ+ groups associated with the misconception that monkeypox is transmitted through homosexual contact [26]. At the same time, conventional means such as television and public infographics should still maintain the role of an official and trusted standard-setting platform to avoid misinformation. Second, financial support schemes, such as discounts during the first three months of vaccination launch, can be provided to help citizens transition from free-of-charge COVID-19 vaccines to compulsory and with charge monkeypox, as well as to promote early vaccination among the public.

The main strength of our study is the complexity of the DCE scenarios that we provided to respondents and the geographical variation. However, a limitation was that as monkeypox is a recently emerged health issue after a long period of COVID-19, many participants may be influenced by their previous perception of COVID-19 when answering specific questions, such as willingness to pay for vaccines. Lastly, data collected through self-reporting may be subject to bias, and we did not survey a representative sample of the general population. For instance, our sample was made up of a majority of women, people with higher education, and students. This trend is attributed to the fact that our survey was distributed online and that participation was voluntary, meaning people who are more conscious of their health or engage in online content more frequently would be more likely to respond to our survey. Nonetheless, this study is among the first and most significant efforts to examine national knowledge of monkeypox and provides early insights on how monkeypox vaccination and management should proceed in the coming stage. Future research direction can be on the optimal price and discount level, cost distribution strategy across different socioeconomic and geographical groups, social factors and other factors related to the COVID-19 isolation period that influence the decision to vaccinate.

## CONCLUSION

Our study presented lower-than-expected knowledge of monkeypox among Vietnamese respondents. Although the monkeypox vaccine was widely accepted, the willingness to pay was critically low. As lack of information and misinformation were the primary predictors of vaccine reluctance and unwillingness to pay, urgent and effective information dissemination should be implemented, particularly through social media and television. Regarding vaccination, authorities should prioritize high-risk groups and consider the population's willingness to be vaccinated to determine the optimal vaccine price without compromising the country's economic status and limited health resources.

## Additional material


Online Supplementary Document
