# Supplementary material for: Crucial choices in a global health crisis: Revealing the demand and willingness to pay for a hypothetical monkeypox vaccine – the PREVENT study
Source: J Glob Health. 2023 May 5;13:04033. doi: 10.7189/jogh.13.04033 (PMC10161964; doi:10.7189/jogh.13.04033)
Supplement: Online Supplementary Document [file jogh-13-04033-s001.pdf]

SECTION 1: Development of the Vietnamese PREVENT's research tool

Outcome variables

Willingness to take a new hypothetical vaccine against Monkeypox

We utilized a short questionnaire to measure the willingness to take a hypothetical new Monkeypox vaccine using the following four options:

1) Will not vaccinate
2) Not decided yet, waiting for more information
3) Will vaccine once vaccine becomes officially approved
4) Ready to vaccinate in the vaccine trial

Willingness to pay for the vaccine

To measure the willingness to pay for the new Monkeypox vaccine, we used a question with five levels: 1) Unwilling to pay; 2) 20% of the cost; 3) 50% of the cost; 4) 80% of the cost; and 5) Full cost. Participants do not know the actual cost of the vaccine. This question provided more information on financial support/discounts for suppliers.

Covariates

Socioeconomic status

Respondents reported that questions about their socio-demographic information included age, gender (male, female), marital status (single/divorced/widowed, married), occupation, monthly household income per capita, and area.

History of COVID-19 infection and vaccination

Respondents reported that questions about personal and family history of COVID-19, time since the last COVID-19 infection, health status after infection (Completely healthy: no changes after infection, Relatively healthy: slight decrease in overall health including stamina, memory, Compromised/with illness: serious post-COVID conditions, deteriorating quality of life), and personal and family history of COVID-19 vaccination.

Factors affecting intention to vaccinate for disease prevention

To measure the factors affecting intention to vaccinate for disease prevention of participants, we conducted 10 items corresponding to 3 domains as follows:

Concerns about the impact and composition of the vaccine

1. Concerned that the vaccine is newly developed
2. Concerned about immediate side effects of the vaccine
3. Concerned about long-term side effects of the vaccine
4. Concerned about new components of the vaccine
5. Concerned about the immunity duration of the vaccine

Fear of vaccine

6. Fear of vaccines and injections in general
7. Fear of insufficient information to make an informed decision
8. Wait for others to vaccinate first

Responsibility to the community

9. Vaccinate so that the community can maintain normal living and working conditions
10. Vaccinate to fulfill personal responsibility of disease prevention

Interpersonal factors

We utilized 8 items to measure the interpersonal factors of respondents as follows:

Risks of infected diseases

1. How is the risk of reinfection for you and your family members?
2. How is the risk of reinfection for children in your family?

Fear of the impact of the disease on health and economy

3. How afraid are you of the spread of this disease?
4. How afraid are you of the impact on your health of this disease?

5. How afraid are you of the impact on the economy of this disease?

Service Satisfaction

6. How satisfied are you with vaccination services?

7. How satisfied are you with COVID-19 consultation services?

8. How satisfied are you with healthcare services for pandemics?

Knowledge and perception of Monkeypox

We utilized 15 items to measure knowledge and perception of Monkeypox. There were 3 domains:

Factor 1: Incorrect general knowledge about Monkeypox

1) Monkeypox only exists in Western and Middle Africa.

5) Monkeypox is spread through sexual means.

15) Strengthening personal hygiene and nutritional plan as preventative measures.

Factor 2: Knowledge of transmission routes and ways to prevent Monkeypox

2) Vietnam is at risk of developing a Monkeypox outbreak.

3) Monkeypox is spreading fast globally.

6) Monkeypox is spread through skin contact and droplets.

7) Monkeypox has the same manifestation as smallpox.

8) Having multiple pustules is a symptom of Monkeypox.

9) Fever, headache, and swollen lymph nodes are symptoms of Monkeypox.

10) Monkeypox can result in fatigue and death.

12) Not eating wild animals is a preventive measure.

13) Reducing interactions with wild animals is a prevention measure.

Factor 3: Incorrect knowledge about the route of transmission and treatment

4) It is difficult to spread Monkeypox among humans.

11) Treatment for Monkeypox is available.

14) Monkeypox is not transmitted through handshakes, hugs, and kisses.

SECTION 2: Sample size calculation

Sample size for DCE: was determined using the Sawtooth Software used to determine the number of responses: Sample size = (multiplier*c)/(t*a) = 357.

Where: multiplier = 1000.

c = largest number of levels across all features (=5)

t = number of tasks or questions (=7)

a = number of alternatives or choices per question (=2).

The minimum sample size to apply the DCE method was 357 people.

We aimed to recruit a sufficient sample size for each target group, including health workers and general population, because the implications of findings are different for each group.

Finally, this survey had 842 participants. Thus, this sample size was reached for the target of the survey.
